# Can we trust tumour markers in pregnancy after breast cancer? A case of elevated CA 15-3 in the third trimester of pregnancy normalising after delivery

**DOI:** 10.3332/ecancer.2019.979

**Published:** 2019-11-26

**Authors:** Barbara Buonomo, Stefania A Noli, Alessandra Santini, Carlo Alviggi, Fedro A Peccatori

**Affiliations:** 1 Gynecology and Obstetrics Unit, Department of Neuroscience, Reproductive Sciences and Dentistry, School of Medicine, University of Naples Federico II, 80138 Naples, Italy; 2 Fertility and Procreation Unit, Division of Gynecologic Oncology, European Institute of Oncology IRCCS, 20141 Milan, Italy; 3 Department of Clinical Sciences and Community Health, Università degli Studi di Milano, 20122 Milan, Italy; 4 Oncologia Clinica, Azienda Ospedaliera Universitaria (AOU) Sant’Anna, 44124 Ferrara, Italy

**Keywords:** breast cancer, pregnancy, markers, CA 15-3

## Abstract

We realized a narrative review of the current literature starting from the case of a patient with raised CA15-3 during an uncomplicated pregnancy after breast cancer.

The aim of our paper was to assess specificity, physiological changes and clinical utility of CA 15-3 monitoring during pregnancy after breast cancer, starting from clinical practice and retrieving the most relevant evidence in the literature.

## Introduction

Breast cancer is the most common malignant disease diagnosed during the reproductive age, with around 6% of cases occurring in women under 40 years [1]. In this age group, growing attention is given to fertility issues; thanks to improved prognosis and to the availability of fertility preservation strategies, many women can become pregnant after breast cancer treatment [2]. Tumour markers (TM) assessment is not recommended in the follow-up of breast cancer patients by many clinical guidelines [3, 4]; however, the practice of dosing tumour-associated antigens in serum is widely used in clinical practice, including the follow-up of breast cancer survivors during pregnancy. In cancer patients, the increase of TM is mainly caused by the damage of the basement membrane by infiltrating lesions and metastases [5]. Nonetheless, TM levels may change according to renal and hepatic function, inflammation, molecular weight, characteristics of the protein and half-life [6].

CA 15-3 is a high molecular weight (>270 kDa) mucin-type glycoprotein extensively used as TM for breast carcinoma [7]. It has been traditionally considered useful to monitor the response to treatment in metastatic disease as well as in the surveillance of cancer survivors, to detect cancer recurrence [8]. CA 15-3 may increase in many non-malignant conditions including liver cirrhosis, tuberculosis, benign breast disease, pelvic inflammatory disease, endometriosis and autoimmune diseases as systemic lupus erythematosus [9].

The aim of our paper was to assess specificity, physiological changes and clinical utility of CA 15-3 monitoring during pregnancy after breast cancer.

## Methods

Starting from the real case of a patient with raised CA 15-3 during an uncomplicated pregnancy after breast cancer, we performed a narrative review of the current literature.

We searched on PubMed for relevant papers published up to 2nd September 2019, with restriction to publications in English and data in human pregnancy. We used the following keywords: breast AND (carcinoma OR cancer OR neoplasm OR tumor OR tumour), AND (pregnancy OR pregnant OR gestation), AND (tumor marker* OR tumour marker*) AND (CA15.3 OR CA15-3 OR CA15 3 OR carbohydrate antigen 15.3 OR carbohydrate antigen 15-3 OR carbohydrate antigen 15 3). The research produced 194 references. A total of 14 full-text articles and 7 abstracts were deemed eligible and included in this review.

## Clinical case

A 35-year-old woman presented with a right breast lump in August 2014. She underwent quadrantectomy with a pathological diagnosis of moderately differentiated ductal invasive carcinoma (G2), pT1c pN0, oestrogen receptor (ER) 90%, progesterone receptor (PgR) 80%, Ki-67 35%, HER2+++. Staging procedures showed no evidence of metastases. The patient received adjuvant chemotherapy with cyclophosphamide and epirubicin (EC) for four cycles followed by weekly paclitaxel for 12 weeks (wT) concomitant to trastuzumab (T); T was completed after 12 months of therapy, according to the clinical practice, with no relevant toxicity. The adjuvant therapy also included radiation therapy. Hormone therapy was performed with tamoxifen, 20 mg daily with monthly triptorelin until October 2016.

The patient interrupted endocrine treatment to attempt pregnancy in January 2017. After 13 months, a spontaneous pregnancy occurred, which was closely monitored. Amniocentesis revealed a normal foetal karyotype and the ultrasound screening showed no signs of malformations and no other foetal, placental and amniotic fluid anomalies. The patient underwent a regular surveillance follow-up for breast cancer: clinical and serological findings were normal until 4th October 2018 (at 33 weeks + 3 days of gestation) when a raised CA 15-3 level was detected (79.5 U/mL, normal limits < 30 U/mL). Carcinoembryonic antigen (CEA), CA125 and CA19.9 were in the normal range. The subsequent CA 15-3 value was 92.6 U/mL on 15th October 2018.

Delivery after induction of labour with prostaglandins was performed at the 37th week of gestation on 24th October 2018. Vaginal delivery was uncomplicated, with the birth of a healthy baby. One week after delivery, the total body CT scan resulted negative and the CA 15-3 value was normalised. Breastfeeding occurred only from the left gland. At present, both the baby and the mother are in good condition health-wise.

## Literature review

Limited and conflicting data are available about serum CA 15-3 levels throughout normal and complicated human pregnancies. In some studies, CA 15-3 concentrations were significantly lower in non-pregnant women compared to those who were pregnant. The levels increased significantly with the progress of pregnancy even if they rarely went above cut-off values [10–12]. Cheli *et al* [13] evaluated maternal serum levels of four different tumour-associated antigens during the three trimesters of pregnancy in 90 healthy women. They reported that CA 15-3 values were above the cut-off (3.3%) and they were significantly higher in the third trimester as compared to the first trimester of pregnancy (*p* < 0.05).

Other authors concluded that CA 15-3 levels are not influenced by gestation and found no significant difference in serum CA 15-3 concentrations among the three trimesters, remaining a reliable TM for breast cancer during pregnancy [14–16].

Maternal serum CA 15-3 values seem to be significantly higher in primigravida compared to multigravida women and no significant foetal sex-related difference was found [17].

Bon *et al* [18] investigated maternal serum CA 15-3 concentrations both in complicated and physiological pregnancies. They collected serum samples from 120 women, whose pregnancy outcome was pathological (i.e., miscarriage, foetal death, intrauterine growth restriction, chromosomal and structural foetal abnormalities and preeclampsia) and they compared these values with CA 15-3 levels of 350 women with a normal pregnancy. In accordance with previous studies [10–13], they confirmed that CA 15-3 maternal serum levels are higher during the third trimester (median 26.0 U/mL) compared to the first and second one (median 14.0 and 15.0 U/mL; *p* < 0.0001). They found no correlation between CA 15-3 levels and the presence of pregnancy complications as they observed wide fluctuations of CA 15-3 values in pathological as well as in normal pregnancies [18].

Sharma *et al* [19] reported that CA 15-3 concentrations were higher among women with gestational diabetes, intrahepatic cholestasis of pregnancy or heart disease than among those without any complications.

Hegab *et al* [20] compared circulating CA 125, CA 19-9, CA 15-3 and CEA levels between 60 patients with hydatidiform mole and 20 pregnant women with the corresponding duration of pregnancy. No significant statistical difference was found. The serum CA 15-3 levels before and after molar evacuation were within the normal range.

Other groups tried to identify a relationship between TM levels and chromosomal anomalies. Akinlade *et al* [21] reported that CA19-9 was significantly increased in pregnancies with trisomy 21 (0.98 MoM in euploid, 1.16 MoM in trisomy 21, *p* = 0.024), while CA 15-3 did not differ significantly (1.03 MoM in euploid, 1.09 in trisomy 21, *p* = 0.130) as reported by others [22].

Liang *et al* [23] found that serum CA 15-3 levels detected by Ma695–Ma552-based assay was abnormally and significantly higher both in pregnant and in lactating women than in non-pregnant women. This study suggests caution when interpreting the results, taking into account what kind of laboratory assay was used.

Table 1 summarises the reported results, excluding those of the only two available reviews of the literature [6, 24].

## Discussion

Despite clinical guidelines not recommending the use of CA 15-3 for clinical monitoring of patients with a history of breast cancer [3, 4], this TM is still assessed in many patients. Actually, serum levels of CA 15-3 are increased in about 80% of women with metastatic breast cancer [6]. The increase of CA 15-3 in pregnant women with a past history of breast cancer should be carefully evaluated, as misinterpretations may occur. Nowadays, the oncofoetal origin of CA 15-3 is still debated. Most of the studies reported very low CA 15-3 levels in amniotic fluid throughout pregnancy compared to its higher values in maternal serum [6, 14, 17, 25]. These data indicate that this antigen is not produced by foetus, placenta or decidual tissue. Hence, CA 15-3 values should not be considerably influenced by gestation [14–16]. Nevertheless, in our case report, an increase of serum CA 15-3 above the cut-off value appeared during the late third trimester of an otherwise physiological pregnancy: the highest reported CA 15-3 value was 92.6 U/mL. Immediately after delivery, TM level normalised and the total body CT was negative for oncological disease. This is in agreement with the results of most other studies: CA 15-3 values were significantly higher in the third trimester when compared with those in the first and the second trimesters of pregnancy, although they commonly remained within the normal range [11, 12]. Only 3 papers, of 14 considered here, reported CA 15-3 values above the cut-off [13, 15, 16]. Moreover, our research confirmed the results of the systematic review by Han *et al* [24] which observed that CA 125, CA 15-3 and squamous cell carcinoma antigen levels were elevated during normal pregnancies, whereas anti-Müllerian hormone, inhibin B and lactate dehydrogenase levels were below the physiological range. Compared to Han *et al* [24], we extended our review of the literature to those articles including also complicated pregnancies [10, 18–22, 25]: no significant difference in serum CA 15-3 values between normal and pathological pregnancies emerged, except for one paper by Sharma *et al* [19] which showed an increase of CA 15-3 in pregnancies complicated by gestational diabetes, intrahepatic cholestasis and heart disease.

## Conclusions

When high levels of CA 15-3 are detected in the setting of pregnancy after breast cancer, a standard staging approach should be pursued, together with proper considerations on those expected rise of the marker observed in some specific conditions. The current literature on this topic is still controversial and a specific range for ‘normal’ values in pregnant women has not been established yet. Another possible confounder is the lack of a standardised assay for the assessment of CA 15-3 concentrations. Further studies are needed to clarify the real value of TM in determining disease status in pregnant cancer patients.

## Conflicts of interest

The authors declare that they have no conflicts of interest.

## Funding statement

No funding was received for this specific research.

## Authors’ contributions

All authors listed have contributed sufficiently to the writing and/or critical revision of the paper; they have approved the submitted final version.

## Figures and Tables

**Table 1.** Summary of the reported papers on CA15-3 levels during normal and pathological pregnancies.

Authors/year of publication	Pathological conditions of pregnancy	Sample	Number of patients	Cut-off value (U/mL)	Results
Touitou *et al*, 1989 [11]	None	MS	- 100 healthy pregnant women - 22 healthy non-pregnant volunteers	<25	Major increase during the third trimester; none above cut-off value.
Lelle* et al*, 1989 [25]	Pre-eclampsia	UC, MS and AF	134 pregnant women at 7–43 weeks of gestation (3/134: twin pregnancies; 5/134 pre-eclampsia)	£25	9.4% of CA 15-3 values were above the cut-off during gestation. Not detectable in UC. Low in AF with a range of 2–18 U/mL.
Schrocksnadel *et al*, 1993 [10]	Gestational hypertension	MS	- 50 patients with gestation hypertension - 50 healthy pregnant patients - 50 healthy non-pregnant controls	£25	CA 15-3 lower in non-pregnant con­trols (median < 5 U/L) than in healthy pregnant women (median 21 U/L) (p < 0.0001), but similar in healthy and hypertensive pregnant women (median 18 U/L) (*p* = 0.34)
Noci* et al*, 1995 [22]	Down’s syndrome	AF	- 20 single Down’s syndrome pregnancies at 15–19 weeks - 60 controls	NR	The median MoM values of CA 15-3 in Down’s syndrome pregnancies were 1.16 MoM, not significantly different from those of unaffected pregnancies (CA 15.3: 0.99 MoM).
Schlageter *et al*, 1998 [12]	None	MS	12 healthy pregnant women at 6–40 weeks of amenorrhoea	£30	Linear temporal evolution of CA1 5-3 but with concentrations in the usual range of values.
Tayyar *et al*, 1999 [17]	None	AF and MS	62 pregnant women at 16–20 weeks of gestation	NR	MS CA 15-3 values were elevated in the primigravida group.
Cheli *et al*, 1999 [13]	None	MS	90 healthy pregnant women during the three trimesters of pregnancy	NR	CA 15-3 values were above the cut-off (3.3%) and were significantly elevated in the third trimester as compared to the first trimester of pregnancy (*p* < 0.05).
Botsis *et al*, 1999 [16]	None	AF and MS	- T1 = 20; - T2 = 29; - T3 = 26; - at parturition = 20; - controls: 20 healthy, age-matched, non-pregnant women	£33	CA 15-3 values in AF, which were marginally higher than in MS, did not differ significantly with the progression of pregnancy. 5%, 10% and 20% above cut-off value, in the three trimesters, respectively.
Bon et al, 2001 [18]	Spontaneous abortion, foetal death, intrauterine growth retardation, chromosomal abnormalities, (pre)-eclampsia and structural abnormalities	MS	- 350 normal pregnancies (T1 = 127, T2 = 192, T3 = 47) - 120 pathological pregnancies (spontaneous abortion = 8, foetal death = 8, intrauterine growth retardation = 46, chromosomal abnormalities = 12, (pre)-eclampsia = 6 and structural abnormalities = 42)	NR	MS CA 15-3 levels in normal pregnancies were significantly higher during the third trimester compared to the first two trimesters of pregnancy. Serum CA15-3 levels in the serum of pregnant women with a pathological outcome were not significantly different from those found in normal pregnancy.
Hegab *et al*, 2003 [20]	Complete hydatidiform mole	MS	- 60 cases of complete hydatidiform mole - Controls: 20 therapeutic abortion of a corresponding duration of pregnancy	NR	No significant statistical difference was found between all groups.
Kiran *et al*, 2005 [14]	No	UC and MS	53 pregnant women just before caesarean delivery of full-termed pregnancies (38–42 weeks)	£30	MS levels of CA 15-3 are not influenced by pregnancy. UC CA 15-3 levels were significantly lower than MS levels at term pregnancies.
Ercan *et al*, 2011 [15]	No	MS	30 healthy pregnant women	£25	It was found that the three trimesters had statistically similar levels for serum CA 15-3 (median values 17.5, 19.7 and 18.3 U/mL, respectively). CA 15-3 assay values were found generally within the normal range. Fourteen patients’ CA 15-3 values were elevated above the cut-off value of 25 U/mL (16%) during each trimester: T1 = 5; T2 = 5; T3 = 4.
Akinlade *et al*, 2012 [21]	Trisomy 21	MS	- 69 trisomy 21 - 388 euploid controls	NR	Not affected by gestational age. No evidence of a significant difference in CA 15–3 concentrations among trisomy 21 and control pregnancies (1.03 MoM in euploid, 1.09 in trisomy 21, *p* = 0.130)
Sharma *et al*, 2015 [19]	Gestational diabetes, preexisting heart disease, intrahepatic cholestasis of pregnancy, thyroid disorder, anaemia, coagulation disorder, hypertensive disorder, epilepsy	MS	- 31 non-pregnant women - 251 pregnant women: 77/251 (30.7%) with high risk pregnancies: gestational diabetes = 20 (26.0%) preexisting heart disease = 16 (20.8%) intrahepatic cholestasis of pregnancy 13 (16.9%) thyroid disorder = 11 (14.3%) (9 hypothyroidism and 2 hyperthyroidism) anaemia = 11 (14.3%) coagulation disorder = 9 (3.6%) hypertensive disorder = 9 (3.6%) epilepsy = 3 (3.9%)	£30	Among the pregnant women, 93 (37.1%) had a CA 15-3 value above the normal cut-off. CA 15-3 concentration was significantly higher in the second and third trimesters than in the first trimester (*p* ≤ 0.01 for both comparisons). CA 15-3 level was also increased in pregnancies associated with gestational diabetes mellitus, intrahepatic cholestasis of pregnancy and heart disease.

NR, not reported; MS, maternal serum; AF, amniotic fluid; UC, umbilical cord; T1, first trimester; T2, second trimester; T3, third trimester
